# The "giant dog ear" sign of left atrial appendage aneurysm—revisited on 3 T cardiac MRI (free-breathing, non-contrast)

**DOI:** 10.1259/bjrcr.20150292

**Published:** 2016-10-22

**Authors:** Alpa Bharati, Suleman Merhcant, Chinmay Nagesh, Ashank Bansal

**Affiliations:** ^1^ NM Medical Centre, Bandra, Mumbai, India; ^2^ Department of Radiology, LTMMC and LTMGH, Sion, Mumbai, India

## Abstract

Left atrial appendage aneurysm (LAAA) is a rare congenital anomaly, usually identified incidentally on a chest radiograph performed for another indication. Our case is that of an 11-month-old male infant who was incidentally diagnosed as having a giant LAAA while being clinically evaluated for pneumonia. The lesion was accurately diagnosed on preoperative, non-contrast, free-breathing cardiac MRI (CMR). LAAA has a peculiar appearance, resembling a “giant dog ear”—a sign first described on cardiac angiography and holding true on CMR as well. Fast free-breathing sequences on CMR, especially on 3 T, provide high-resolution images and eliminate the need for other pre-operative imaging that are either invasive, involve radiation exposure, require general anaesthesia or a combination of these.

## Introduction

Left atrial appendage aneurysm (LAAA) is a rare congenital malformation, usually discovered incidentally in adults. An abnormal cardiac silhouette on a chest radiograph usually prompts further evaluation. Cardiac MRI (CMR) is the only non-invasive, non-contrast and radiation-free technique that provides both anatomic detail and also evaluates cardiac function, serving as a one-stop shop. Barring one case of antenatal diagnosis of LAAA, our case of an 11-month-old male is the only other reported case of an infant being incidentally diagnosed with a giant left atrial aneurysm. Our case highlights the imaging appearance of this rare lesion and the huge potential of CMR in presurgical planning, and also our experience of the significant advantages of free-breathing, non-contrast CMR under sedation in infants.

## Case report

**Figure 1.**
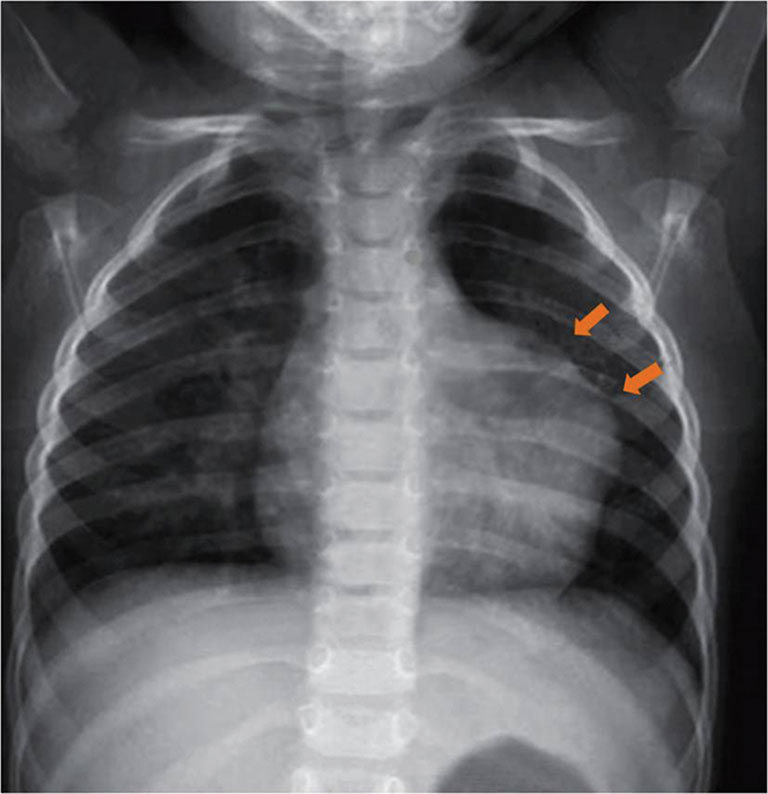
Frontal radiograph of the chest reveals cardiomegaly with a prominent convexity (arrows) of the upper left heart border. The lung fields are unremarkable.

**Figure 2.**
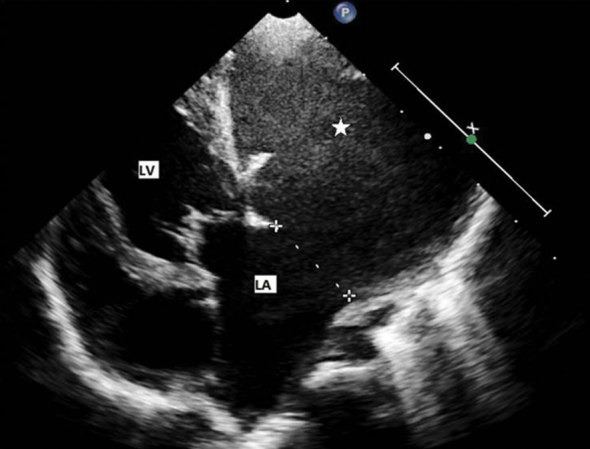
Transthoracic echocardiography (four-chamber view). Echocardiography shows a large left paracardiac lesion (star) widely communicating with the LA. LA, left atrium; LV, left ventricle.

An 11-month-old male infant was admitted with high-grade fever and symptoms and signs of a lower respiratory tract infection. A chest radiograph revealed normal lung fields and an abnormal cardiac silhouette, with a prominent bulge along the left cardiac border (Figure 1). This led to further evaluation by a transthoracic echocardiogram (TTE), which revealed a large left paracardiac blood-filled sac with sluggish flow (Figure 2). This sac occupied most of the field of view on the echo windows, making it difficult to assess the extent of the lesion and its relationship to the left atrium (LA). A CMR was then performed for further characterization of the lesion, in particular to determine its extent and effects on cardiac function.

A non-contrast CMR was performed on a 3 T scanner under oral sedation with chloral hydrate syrup. The scan time was 30 min. After administration of chloral hydrate, the infant was allowed to sleep in his mother’s arms, wrapped in his own blanket. Once sedated, the infant was transferred to the MRI scanner, where vectorcardiographic leads were applied. He was scanned using a 32-channel cardiac coil placed around his blanket. In our experience, it is highly comforting to the child to be examined while wrapped in the same fabric they are used to at home or in the ward. It not only helps sedate them more easily, but also maintains the sedation for the 30–40 min taken to complete the study.

**Figure 3.**
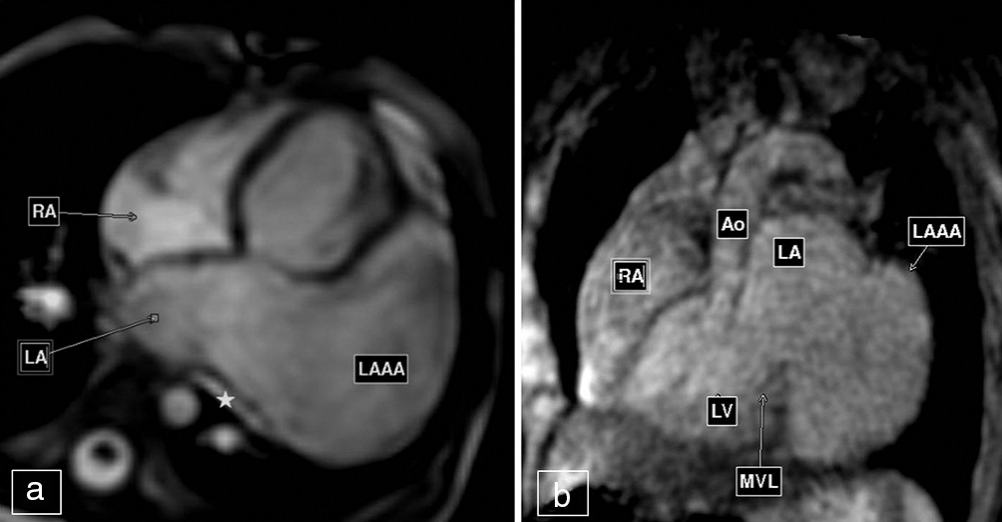
(a) Still images taken from four-chamber cine fast-field echo sequence and (b) reconstructed image from three-dimensional whole-heart sequence. LAAA is seen as a “dog ear” like protrusion. It is adherent to the lateral wall of the basal LV. The left superior pulmonary vein (star) is being compressed by the aneurysm. Ao, aorta; LA, left atrium; LAAA, left atrial appendage aneurysm; LV, left ventricle; MVL, mitral valve; RA, right atrium.

**Figure 4.**
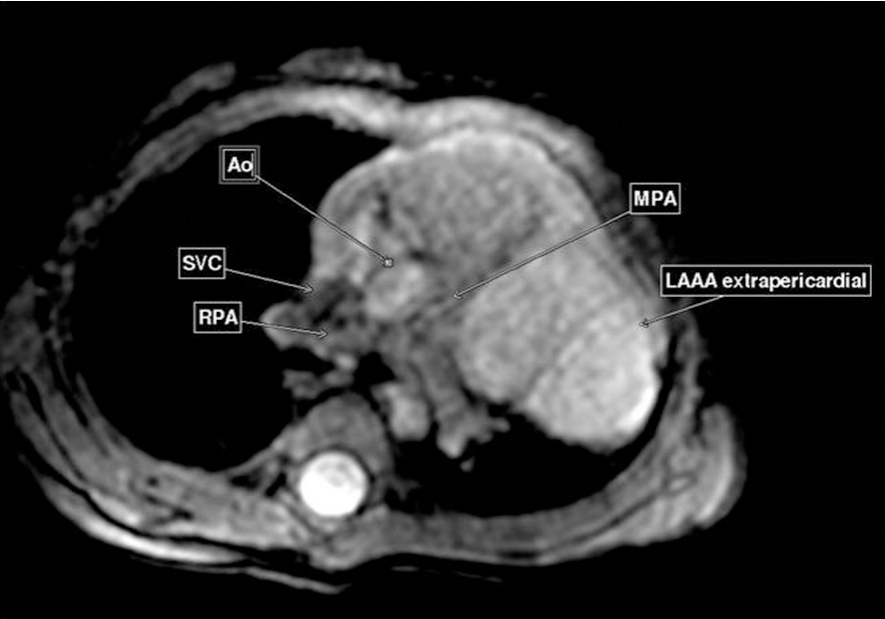
Reconstructed image from three-dimensional whole-heart sequence shows an extrapericardial component of the lesion protruding across the pericardial defect. A septum at the level of the MPA bifurcation probably represents the margin of the pericardial defect. Ao, aorta; LAAA, left atrial appendage aneurysm; MPA, main pulmonary artery; RPA, right pulmonary artery; SVC, superior vena cava.

We obtained axial three-dimensional (3D) whole-heart images using respiratory-gated spoiled gradient (SPGR) sequence and cine images in standard cardiac planes using free-breathing steady-state free-precession (SSFP) sequences. The former provided volumetric data that was reconstructed in various planes to define the location, morphology and extent of the lesion. The latter was used for evaluating the ventricular function and blood flow within the lesion. The CMR depicted a 7-cm large, predominantly intrapericardial dog ear-like protrusion arising from the LA (Figure 3a) with slow swirling flow within (Supplementary Video 1). It was diagnosed as a congenital LAAA compressing the left superior pulmonary vein (Figure 3a). It was seen adhering to the adjoining left ventricle (LV) and causing a mild mass effect on the LV in the form of a flattening of its basal lateral wall (Figure 3b). The superior aspect of the aneurysmal sac showed a small septum, which was suspected to be the site of a pericardial defect owing to a small extrapericardial herniation of the aneurysm (Figure 4). The mitral valve was unremarkable. No other congenital cardiac defect was seen. The biventricular function was normal. The CMR findings were confirmed on complete surgical resection of the aneurysm.

## Discussion

LAAA is a rare congenital condition. It was first described as a “giant dog ear” by Dimond et al^1^ on cardiac angiography. Its clinical presentation ranges from an incidentally detected abnormal cardiac silhouette on chest radiographs to complaints of dizziness, chest pain, dyspnoea and palpitation, often secondary to supraventricular arrhythmias. Occasionally, serious complications such as thromboembolic stroke and congestive cardiac failure have also been reported.^2,3^ Most of these lesions are excised, either to relieve the symptoms or to prevent future complications.

LAAA may be intra- or extra-pericardial. Intrapericardial aneurysms are hypothesized to be due to an inherent atrial wall weakness, while the latter are caused by gradual forceful extrusion of the atrial wall or appendage through a pericardial defect.^3,4^ Intrapericardial aneurysms have an intact pericardium^5,6^ and cause distortion and mass effect on the left ventricle,^2^ as in our case. An intact pericardium is well delineated on a *T*_1_ weighted sequence, although it can only be confirmed at surgery.^2^

On the chest radiograph, LAAA is seen as a large convex bulge that shows mild-to-moderate extension beyond the left heart border in the position of the auricular arch. The differential diagnoses on chest radiograph would include prominent epicardial fat, pericardial cyst, loculated pericardial effusion, cardiac or paracardiac tumour or a pericardial defect. However, these are easily differentiated on MRI, based on their tissue characteristics.

TTE, often the first study following a chest radiograph, usually identifies a paracardiac vascular lesion, but is of limited utility owing to its small acoustic window. Hence other imaging modalities are often used to confirm the diagnosis for better pre-surgical evaluation. Transesophageal echocardiograpy (TEE), although widely used, is an invasive procedure with the theoretical risk of aneurysm rupture and thrombus dislodgement.^2^ Another limitation of both TTE and TEE is that they may miss the origin of the aneurysm.^6^ Catheter angiography is invasive, involves radiation exposure and contrast usage. Multidetector CT also involves radiation exposure and contrast usage and is unable to assess cardiac function. False-negative angiograms have been reported owing to slow filling of the aneurysm, with resultant dilution of the contrast or owing to thrombosis of the aneurysm.^3,7^

The spatial and temporal resolution in CMR allows for a better assessment of the spatial relationships of the lesion with the surrounding cardiac structures, as well as a detailed evaluation of each cardiac chamber and valvular function. Free-breathing 3D whole-heart imaging allows multiplanar reconstruction and appropriate display of images vital for surgical planning, as was possible in our case on 3 T MRI.

Compared with 1.5 T, 3 T MRI has four times the speed and twice the spatial resolution. The compensation in time may be used to obtain images of similar spatial resolution faster or can be used to obtain higher resolution images in the same time as 1.5 T. In paediatric 3 T CMR, spatial resolution can be traded off for time. As a result, 3 T imaging can potentially facilitate diagnostic quality free-breathing cine images in a much shorter time than can 1.5 T scanners. The main disadvantage of 3 T CMR is the greater sensitivity to artefacts arising from field inhomogeneity. While this can be overcome with adequate shimming and by utilizing frequency scout imaging, it can be technically challenging.^8^

Barring one patient with antenatal diagnosis of LAAA who underwent confirmatory post-natal CMR, our case of an 11-month-old male infant is the only other reported case of an infant being incidentally diagnosed with a giant left atrial aneurysm, a condition usually reported in adults. Our case also emphasizes the utility of free-breathing SSFP and 3D SPGR sequences in this age group during CMR for the assessment of LAAA.

## Conclusions

CMR is an extremely valuable addition to the armementarium of non-invasive diagnostic imaging in congenital heart diseases. It can provide accurate anatomical and functional information for surgical planning and management. In some cases, it may completely eliminate the use of invasive techniques as well as general anaesthesia, as in our case. The description of LAAA as a “giant dog ear” used by Dimond et al^1^ on cardiac angiography holds true on CMR as well. The uncanny resemblance of LAAA to a large dog ear makes this sign a relatively easy way to diagnose this rare lesion.

## Learning points

1. LAAA has a rare differential of a bulge along the left heart border in the position of the auricular arch. The differential on chest radiograph would include prominent epicardial fat, pericardial cyst, loculated pericardial effusion, cardiac or paracardiac tumour or a pericardial defect.
2. Free-breathing SSFP and 3D SPGR sequences on 3 T MRI with the infant wrapped in his/her own blanket can help perform cardiac MR under oral sedation, thus obviating the need for general anaesthesia.
